# Recent Advances of Silver-Based Coordination Polymers on Antibacterial Applications

**DOI:** 10.3390/molecules27217166

**Published:** 2022-10-23

**Authors:** Wenfeng Zhang, Gaomin Ye, Donghui Liao, Xuelin Chen, Chengyu Lu, Alireza Nezamzadeh-Ejhieh, M. Shahnawaz Khan, Jianqiang Liu, Ying Pan, Zhong Dai

**Affiliations:** 1 Guangdong Provincial Key Laboratory of Research and Development of Natural Drugs, School of Pharmacy, Guangdong Medical University Key Laboratory of Research and Development of New Medical Materials, Guangdong Medical University, Dongguan 523808, China; 2 The First Dongguan Affiliated Hospital, Guangdong Medical University, Dongguan 523808, China; 3 Chemistry Department, Shahreza Branch, Islamic Azad University, Shahreza 311-86145, Iran; 4 Department of Chemistry, Aligarh Muslim University, Aligarh 202002, India

**Keywords:** metal-organic frameworks, anti-bacterial effect, anti-bacterial mechanism, silver

## Abstract

With the continuous evolution of bacteria and the constant use of traditional antibiotics, the emergence of drug-resistant bacteria and super viruses has attracted worldwide attention. Antimicrobial therapy has become the most popular and important research field at present. Coordination Polymer (CP) and/or metal-organic framework (MOF) platforms have the advantages of a high biocompatibility, biodegradability, and non-toxicity, have a great antibacterial potential and have been widely used in antibacterial treatment. This paper reviewed the mechanism and antibacterial effect of three typical MOFs (pure Ag-MOFs, hybrid Ag-MOFs, and Ag-containing-polymer @MOFs) in silver-based coordination polymers. At the same time, the existing shortcomings and future views are briefly discussed. The study on the antibacterial efficacy and mechanism of Ag-MOFs can provide a better basis for its clinical application and, meanwhile, open up a novel strategy for the preparation of more advanced Ag-contained materials with antibacterial characteristics.

## 1. Introduction

At present, people’s yearning for a better life makes people pay more and more attention to disease and health. However, pathogenic bacteria have a high rate of morbidity and mortality properties. Various antibiotics are used globally to treat infections resulting from bacteria. This wide use results in the critical resistance of bacteria against the drugs used, super viruses, and the lack of effective treatment methods. Therefore, searching for a new treatment or antibacterial drugs has become the hottest topic [1].

Metal-organic frameworks (MOFs) are fascinating porous coordination polymers (CPs), which are made up of organic ligands and metallic cations or metal-containing nodes [2,3,4,5,6,7,8,9,10,11,12,13,14,15,16,17,18,19,20,21]. Owing to their high intermolecular pores, specific surface area, unsaturated active metal sites, and structural and functional diversities [22,23,24,25,26,27,28,29,30,31,32,33,34,35], they have adopted a critical interest in various fields, such as energy storage, ion exchange, sensors, drug delivery/release, separation, molecular recognition, catalysis and theranostics [36,37,38,39,40,41,42,43,44,45,46,47]. Compared with some traditional materials such as porous zeolite materials, polymers, and other materials [48,49,50,51,52,53,54,55,56], MOFs have a specific surface area and volume, a tunable pore size, and a better biocompatibility [57,58,59], which make them an ideal candidate for antimicrobial activity and cancer therapy, and so on [60,61,62,63,64]. In addition, MOF porosities and compositions can be tweaked by carefully choosing organic components and metal ions to achieve precise physical and chemical features [39,65,66,67,68,69]; and nanoscale MOFs have remarkable loading capacities, which are good for drug loading [39,40,70,71,72,73,74,75,76].

MOFs have both organic and inorganic components to provide anti-virus and sterilization effects, which can save human health related to bacterial contamination [77,78,79,80,81]. Compared with other disinfectants and antibacterial agents, MOFs have critical advantages due to their high durability, long-term persistence, critical efficacy, and thermal and optical stabilities [81]. In synthesizing antibacterial MOFs, a polar-organic solvent, well-soluble salts act as a metallic center source (Zn^2+^, Co^2+^, Cu^2+^, and Mo^6+^), and azo compounds act as organic linkers. The metal cations can be easily tuned for the functional application in the MOF synthesis. The metallic cations mentioned above commonly have a better antibacterial activity and can be easily introduced into the frameworks [82].

**Figure 1:**
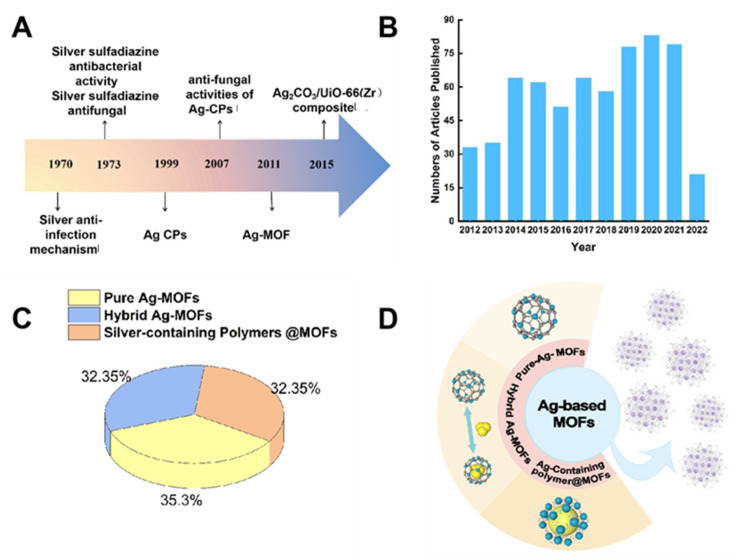
(**A**): Milestones in developing Ag-MOFs for antibacterial activities [89,90,91,92,93,94,95]. (**B**): The trend in the numbers of Ag articles published, 2012–2022. (**C**): The proportion of pure Ag-MOFs, hybrid Ag-MOFs, and silver-containing polymer @MOFs. (**D**): The species of Ag-MOFs.

However, the toxicity of those metal ions has a certain influence on the clinical application. In recent years, the reports on silver-based drugs have also increased, authenticating silver’s role in medical applications. The number of publications related to Ag has significantly increased over the past decade, as shown in (Figure 1B).

Metallic silver has a lower cytotoxicity and immunological response [83], a better stability, and antibacterial properties than other metals, therefore, they are used for drug delivery, medical imaging, and molecular diagnostics [84]. Metallic silver with different shapes and sizes showed great prospects in terms of bacterial infection [85]. Due to the high affinity of Ag to extracellular and intracellular nitrogen and sulfur-containing biomolecules, such as nucleic acids and proteins, common cell activities, such as cell division and respiration would be affected, which eventually causes bacteria death. Ag nanostructures exhibit a size-dependent antibacterial efficiency. The smaller the Ag nanoparticle, the higher the antibacterial efficiency [86]. Although antibacterial efficiency could be improved by controlling the size and surface charges of the Ag nanomaterials, an unavoidable fact is that they tend to aggregate due to their colloidal instability. This aggregation makes silver nanomaterials less capable of entering the bacteria and also decreases the amount of intercellular Ag^+^ [86].

The metal-organic frameworks (MOF) in which the target metal ions can be anchored have attracted the attention of researchers. The metal center in Ag-MOFs, is encapsulated by organic ligands and evenly dispersed throughout the framework [80], allowing the slow and sustained release of metallic species (as cations of natural metal species) to diminish the potential large toxicity caused by the release of the sudden metal ion. At the same time, silver (Ag I)-based antibacterial agents have features of long-acting bacteria with a high stability, a broad antibacterial spectrum, a low volatility, and a low tendency to induce bacterial resistance [87,88]. The key developments of Ag-MOFs for their antibacterial activities are depicted in (Figure 1A) [89,90,91,92,93,94,95].

Despite the wide industrial/medical applications of silver-contained chemicals, as far as we know, no specific antibacterial mechanism of Ag-MOFs has been entirely clarified. A lot of current studies show that Ag-MOFs have an obvious inhibitory effect on Gram-positive and Gram-negative bacteria, *Escherichia coli* (*E. coli*) and *Staphylococcus aureus* (*S. aureus*) [96,97,98,99,100,101,102,103]. The main mechanism of action is due to (1): the high electrical conductivity of metallic silver, the generated static electricity has a strong affinity for sulfur proteins, making silver ions adhere to the cell membrane; (2): the adhesion of silver ions can enhance the permeability of the cytoplasmic membrane and lead to the destruction of the cell membrane; (3): when silver ions enter the cells, they lead to the inactivation of enzymes and the production of reactive oxygen species (ROS). ROS further promote the cell membrane rupture and the DNA replication interruption [81,90,97].

Because of Ag-based MOFs’ excellent performance in antibacterial activity, we have summarized a prospective review of the current developments of Ag-based MOFs (pure Ag-MOFs, hybrid Ag-MOFs, and Ag-containing-polymer @MOFs, Figure 1C,D shows the proportions and types of the three MOFs) for their antibacterial effect. We have also explored the corresponding mechanism and the latest state of art developments in the antibacterial activities of Ag-MOFs. At the end of the manuscript, we envision the challenges and outlook for developing antibacterial Ag-MOFs in future endeavors.

## 2. Silver-Based MOFs

Pure Ag-MOFs refer to materials with a three-dimensional structure, prepared using certain methods (in-situ growth, hydrothermal reaction, etc.) using silver as the metal nodes and organic linkers. Mainly, MOFs are used as “reservoirs” of Ag cations to ensure the controllable release of metal ions. The presence of silver can provide certain antibacterial activities to the MOFs, which is higher than the commercial silver nanoparticles. The MOF system can control the release rate of silver and avoid the toxicity caused by the sudden release of silver. Based on pure Ag-MOFs, the antibacterial effect of MOFs can be increased, or other synergies can be obtained by adding certain modifiers or loading antimicrobial agents. These polymer materials (such as chitosan, polyvinyl alcohol, graphene oxide, etc.) have unique properties (i.e., anticoagulant, high biocompatibility, hydrophilic, antibacterial, etc.). Their addition allows Ag-MOFs to have a more accurate antibacterial activity than the pure Ag-MOFs, and can also improve the antibacterial effect.

Silver-containing polymer @ MOFs mean that on the other MOF structure load, the silver and chemical materials obtain the antibacterial properties or have other special performances. These MOFs usually have some characteristics, such as a high porosity, a strong stability, a good biological activity, they prevent the Ag ions reunion and antibacterial properties, etc., which can help Ag to have a greater antibacterial effect. At the same time, the other MOFs, as the carrier of silver, not only have a certain antibacterial ability, but also can improve the loading amount of silver, and thus improve the release amount of the silver ions.

### 2.1. Pure Ag-MOFs

The three-dimensional structure of the silver-MOFs, Ag_6_(m-O_3_PC_6_H_4_CO_2_)_2_, MOF-**1** has been prepared through the hydrothermal reaction of Ag (NO_3_) and *m*-phosphonobenzoic acid. The organic part is constituted by 3-phosphonobenzoic acid, which is a rigid organic molecule and is classified as a hard base; hence, the silver-MOF-**1**, acts as a reservoir of Ag^+^, which possesses an intermediate stability that exhibits the release of the Ag^+^ ions with the consequent bactericidal effect, and can be utilized against the Gram-positive *S. aureus* (minimum bactericidal concentration (MBC)) value, 50–70 µM) and the Gram-negative strains of *Pseudomonas aeruginosa* (*P. aeruginosa*) (MBC value, 20–30 µM). Furthermore, it was shown that MOF-**1** did not exhibit a significant cytotoxicity [98].

Quaternized carboxylate ligands have a good water dispersibility and stability, and they are used as ligands to prepare 3D Ag-MOF-**2**, [Ag_2_(Cedcp)]_n_, (H_3_CedcpBr denotes *N*-(carboxyethyl)-(3,5-dicarboxyl)-pyridinium bromide), which showed a good stability and solubility, and could release Ag^+^ better, resulting in the strong antibacterial activity towards the Gram-negative and Gram-positive bacteria strains. The Ag-MOF-**2** mainly destroys the bacterial membrane through the synergistic effect of the ligand’s characteristic aromatic ring and positively charged pyridine and the release of Ag^+^, resulting in bacterial death. In addition, the MOF-**2** showed little hemolytic activity on mouse erythrocytes and exhibited an excellent in vitro biocompatibility [99].

**Figure 2:**
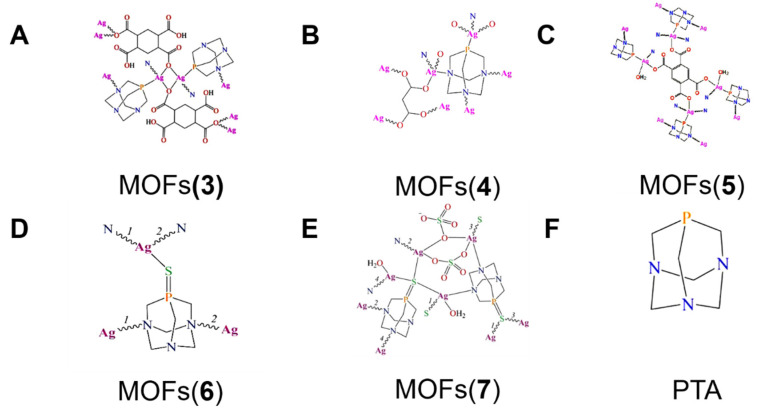
The structural formulas of MOF (**3**) (**4**) (**5**) (**6**) (**7**) and the PTA. Reproduced with permission from [100,101,102,103].

The MOF-**3** (Figure 2A) [Ag_2_(μ_3_-PTA)_2_(μ_2_-chdc)]_n_·5nH_2_O was prepared from 1,3,5-triaza-7-phosphoadamantane (PTA) (Figure 2F) and flexible cyclohexanecarboxylic acid, 1,4-cyclohexanedicarboxylic (H_2_chdc). The 3D structure of the composite material granted their water solubility, air stability, and coordination environment around the silver ion, which was conducive to the release of the biologically active Ag^+^, thus exerting an effective antifungal activity against the Gram-positive *S. aureus* and the Gram-negative *E. coli* and *P. aeruginosa* bacteria and yeast (*Candida albicans* (*C. albicans*)). Among them, it had the strongest inhibitory effect on *S. aureus* and *E. coli*, reaching a minimum inhibitory concentration (MIC) of about 10 and 7 µg mL^−1^ [100].

The pure MOF-**4** (Figure 2B) [Ag_2_(μ_4_-PTA)(μ_4_-mal)]_n_ has been synthesized using PTA as a main fundamental block and flexible aliphatic dicarboxylic acids (malonic (H_2_mal) acids) as an ancillary ligand source. The silver(I) coordination polymers feature a solubility in water and show critical antibacterial and antifungal activities towards the selected strains of the Gram-negative MIC of *E. coli* and *P. aeruginosa* (6 and 7 μg mL^−1^, respectively) and the Gram-positive MIC of *S. aureus* (8 μg mL^−1^) bacteria and yeast (the MIC of *C. albicans* is 30 μg mL^−1^). The mechanism relates to the coordination sphere around each silver atom that controls the compounds’ antimicrobial efficiencies. A slow Ag^+^ ion releasing into the solution would be expected in the presence of an organic ligand containing *O* and *N* atoms as weaker donating centers. This can increase the replacement of organic ligands by the biological moieties to release the Ag(I) cations [101].

The MOF-**5**, (Figure 2C) [Ag_4_(µ-PTA)_2_(µ_3_-PTA)_2_(µ_4_-pma) (H_2_O)_2_]_n_, was assembled from Ag_2_O, PTA, and pyromellitic acid (H_4_pma), which possess a very complex ribbon-pillared 3D metal-organic framework. MOF-**5** acts as a potent antimicrobial agent against the pathogenic strains of the standard Gram-negative *E. coli*, *P. aeruginosa* and the Gram-positive *S. aureus* bacteria, as well as yeast (*C. albicans*). Meanwhile, this MOF also depicted a critical antiviral activity towards the human adenovirus 36 (*HAdV-36*) and exhibited a high cytotoxicity towards an abnormal epithelioid cervix carcinoma (*HeLa*) cell line [102].

Two bioactive Ag–organic frameworks [Ag(u_3_-PTA=S)] _n_(NO_3_) (MOFs-**6**) and [Ag_4_(u_4_-PTA=S) (MOFs-**7**) (Figure 2D,E) (u_5_-PTA=S) (u_2_-SO_4_)_2_(H_2_O)_2_] _n_·2nH_2_O were easily assembled through the 1,3,5-triaza-7-phosphaadamantane-7-sulfide (PTA=S), which can be used as a multifunctional N, S-building block. The coordination modes of PTA=S with different native topologies revealed a significant antibacterial activity against the Gram-negative bacteria, and the MIC values obtained for the MOF-**6** (4–5 µg mL^−1^) were preferentially lower than those of MOF-**7** (20 µg mL^−1^). The observed antibacterial activity of MOF-**6** and MOF-**7** is due to the presence of bioactive silver nodes. The highest antimicrobial activity of MOF-**6** has been reported than that of the corresponding silver MOFs derived from PTA=O, and stronger than other silver(I) salts AgNO_3_ (Figure 2) [103].

Two Ag-MOFs, Ag_5_(PYDC)_2_(OH) (MOF-**8**) and [Ag_2_(O-IPA)(H_2_O)·(H_3_O)] (MOF-**9**), were synthesized using hydroxyl and pyridyl-contained aromatic-carboxylic acids as ligands (HO-H_2_IPA = 5-hydroxyisophthalic acid and H_2_PYDC = pyridine-3, 5-dicarboxylic acid). Utilizing the MOFs as a carrier, the Ag^+^ in both compounds diffuse to the bacterial surface and release the Ag^+^ ions, and the ion channels are destroyed. The Ag^+^ ions may interact with the thiol groups of protein, which can inactivate vital enzymes and disrupt the bacterial membrane’s integrity and permeability. In addition, a functional group of organic ligands in the MOFs can bond with the cations in the cell, such as Ca^2+^ and Mg^2+^. The reaction-oxygen generated in the cell results in the modifications and fragmentation of DNA. It showed a high antibacterial activity against the Gram-negative bacteria (*E. coli*) and the Gram-positive bacteria (*S. aureus*). Furthermore, it has a good biocompatibility with blood cells. The relevant data show that the MIC of the MOFs synthesized by this method is 5–15 ppm for *E. coli* and 10–20 ppm for *S. aureus*, and MOF-**8** and MOF-**9** exhibit diameters of the inhibition zones (ZOI) in 17.0 and 20.0 mm for *E. coli*. The diameters of the inhibition zones against *S. aureus* for MOF-**8** and MOF-**9** are 14.0 and 16.0 mm, respectively, and they are larger than the Ag-NPs [104].

The MOF-**10**, [AgL]_n_·nH_2_O, (L=4-Cyanobenzoate) exhibits a good antibacterial performance against the *S. mutans (UA159)*, *F. nucleatum* (*ATCC 10953*, *Fn*), and *P. gingivalis* oral bacteria. The bactericidal ability is enhanced as the concentration of the metal ions increases. Compared with the traditional antibacterial materials, such as silver nitrate (AgNO_3_), the MOF-**10** dispenses better slow-release bactericidal properties. The sterilization property of the MOF containing the Ag^+^ ions is better than that of the MOF with Zn^2+^ ions [105].

The metal nodes of the Ag^+^ ions and the linkers made of redox-active naphthalenediimide (NDI) derivatives could act as excellent radical-doped Ag-MOF antibacterial materials, such as, the two reported MOFs, such as MOF-**11** Ag (NDI-1)_0.5_(H_2_O) and MOF-**12** Ag_7_(NDI-2)_1.5_(CH_3_S)_4_(DMSO)_3_(DMSO). Because of the synergistic antibacterial effects of the Ag^+^ ions and the reactive organic radicals, those MOFs displayed a broad-spectrum antimicrobial activity against the multi-drug-resistant bacteria; the reported inhibiting rate was more than 98.74%. The colony inhibition ratio (IR) of the MOF-**11** and MOF-**12** against the Gram-negative and Gram-positive strains is about 100% and 99.90%, respectively, which is higher than AgNO_3_ (IR value: 89.29–94.92%) [106].

The Ag-MOFs types, the antibacterial activity against the different bacteria, and their experimental conditions have been tabulated in Table 1. The pure Ag-MOFs were prepared using silver ions as a metal center and the relevant organic systems; the organic ligands effectively wrap the active sites of the Ag metal ions in the framework. The organic ligand is evenly distributed across the overall material, enabling a sustained release of the metal ions to avoid the toxicity caused by a burst release of the metal ions. The Ag-MOFs showed a high antibacterial activity against the Gram-positive and Gram-negative bacteria.

The MOFs-**3**, **4**, **5**, **6**, and **7**, constructed by the linkers 1,3,5-triaza-7-phosphaadamantane (PTA) or 1,3,5-triaza-7-phosphaadamantane-7-sulfide (PTA=S), which possess the feature of water-solubility and air-stability, endow the Ag-MOFs with a better bioavailability, and physiological media by achieving the lowest MIC values and a very high ZOI. As a compatible polymer of the MOFs, PTA can synthesize three-dimensional topology structures and slowly release the Ag^+^ ions into the solution due to the *O* and *N*-donor ligands exerting only a weak binding affinity for the silver, increasing the probability of the organic ligand replacement by the biological moieties. Moreover, the Ag-*O* and Ag-*N* bonds have a better biocompatibility than the Ag-*S* and Ag-*P* bonds [94,107,108]. These characteristics are favorable for the Ag-MOFs to release their silver ions, and then, with their breaking ability to balance the ions and disrupting the integrity of the cell membrane, with the cell internalization of the Ag^+^ ions, they interact with the fragmentation of DNA. At the same time, the structural changes, such as a 3D network, can give the MOFs a higher stability and be more conducive to the Ag^+^ ion antibacterial activity. The 3D structure can also act as a depository for the Ag^+^ cation, ensuring the Ag’s slow and continuous release.

The strong antibacterial activity of the Ag-MOFs is correlated to the donor site of the weak ligand, their binding force, and the synergistic effect of the ligand. The presence of the organic ligands can increase the stability of the silver ions and thus enhance the antibacterial activity. The MOFs-**11** and **12** have a very high and significant effect on the inhibition of the Gram-positive and Gram-negative bacteria, and the inhibition rate (IR) is more than 99.5%. The organic ligand naphthalenediimid (NDI)) of MOF-**11** and MOF-**12** have stable radical-doped coordination compounds that produce organic radicals by unconventional lone pair-Π interactions and generate the synergistic antibacterial effects of the Ag^+^ ions and the reactive organic radicals [109].

The released silver ions are known as the main reason for the antibacterial activity of the pure Ag-MOFs. The main purpose is to release the silver ions from the MOF structure, whether it is the weak binding force between the ligand and silver metal or the synergistic antibacterial action with silver, which is conducive to a series of reactions of Ag^+^ ions on the bacterial membrane and changes the surrounding environment of the cells, and then destroy the ion channels. More importantly, the silver ions react with the thiol groups and the intracellular substances, failing the cell transcription and replication, which ultimately ends with the death of the bacteria.

### 2.2. Hybrid Ag-MOFs

**Figure 3:**
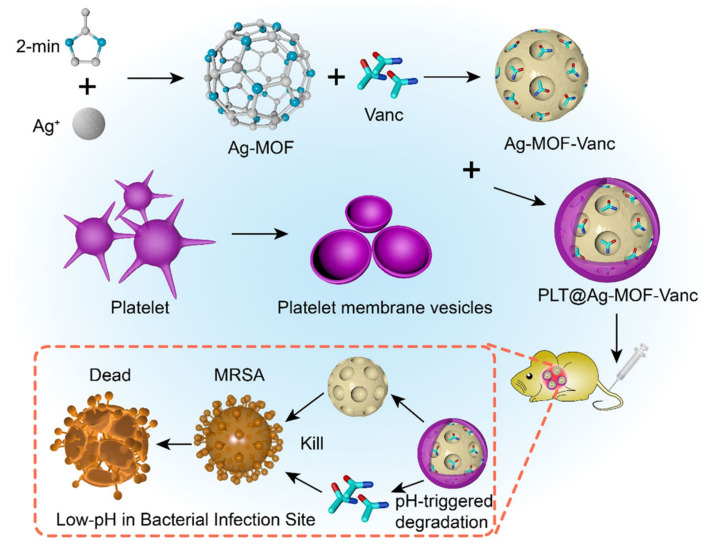
Schematic diagram of PLT@ Ag-MOF-Vanc in the treatment for a MRSA infection. Reproduced with permission from [111].

Huang et al. designed a novel platelet membrane-camouflaged material PLT@ Ag-MOF-Vanc (MOF-**13**). The MOF-**13** can release silver ions and drugs through the pH regulation, avoiding a premature drug release in the circulatory system. The MOF-**13** showed an obvious inhibitory effect on *MRSA*, the MIC of MOF-**13** was 0.5 µg mL^−1^, and the effect was better than with the Ag-MOF-Vanc and vancomycin alone. The antibacterial mechanism may be related to the synergistic reaction of physics and chemistry, which includes targeting *MRSA* via PLTm; the intracellular bacterial metabolism interfering effect; the catalytic effect in the ROS production; the damaging of the cell membrane integrity; and the inhibiting effect on the formation of a biofilm [110,111] (Figure 3).

Using silver acetate as the metal source and 2-aminoterephthalic acid as the organic linker, a new MOF-**14** with a higher antibacterial effect, namely Ag-MOF@TFN (thin-film nanocomposite) was synthesized. The MOF-**14** can completely inactivate and degrade *E. coli* and *S. aureus*, and attributed to the mortality rates of approximately more than 90% of materials embedded within the polyamide matrix and forwarding osmosis membranes, to improve their antifouling and antibacterial properties. While the most probable antibacterial mechanism is related to the release of the Ag^+^, other possible antibacterial mechanisms proposed for the Ag-MOFs’ biocidal properties are (i) direct attachment to the bacterial cells, infiltration, and the physical destruction of the cell membrane and (ii) the indirect generation of the reactive oxygen species which trigger damage to the bacteria cell structure [112].

In an article, S. Fatemeh Seyedpour et al. reported a new antifouling TFC (thin-film composite) polyamide membrane by surface anchoring via the in-situ assembly of the silver-based MOFs. The MOF-**15** (Ag-MOFs @TFC) have antibacterial properties against gram-negative bacteria (*P.aeruginosa strains*), and a bacterial mortality rate (MR) of approximately 100% was attained. The antibacterial mechanism of the Ag-MOFs includes several pathways; (i) direct adherence of the Ag ions to the bacterial cells and permeating into them, (ii) the release of Ag^+^ from the dissolved Ag NPs and then penetration to the bacteria inside, and (iii) the indirect production of reactive oxygen species by the Ag NPs and Ag^+^. In other words, MOFs can be a reservoir of metal ions, such as silver (Ag^+^), and the chronic depletion of the metal ions by the destruction of the framework can create a sustainable antibacterial activity [113].

The Ag-MOFs (Aand Gram-negative bacteria, respectively, and the maximum diameter of the inhibition zone of *S. aureus* and *E. coli* is 12.1 and 9.7 mm, respectively. The MOF-**16** has an anticoagulation effect and a good biocompatibility because of the presence of PVA and CS. The MOF-**16** can release Ag^+^ to strongly attract the enzyme protein in bacteria and quickly bind together to destroy the bacterial cell membrane. The Ag^+^ can also form a reactive oxygen species (ROS) that further attacks the cell membranes [114].

The MOF-**17** (Ag-MOF−CQDs) were synthesized by the nanocomposites and g_5_(PYDC)_2_(OH)) (PYDC=Pyridine-3, 5-Dicarboxylic Acid) was synthesized under the modified hydrothermal, and then the polyvinyl alcohol (PVA) hydrogel modified the Ag-MOFs as the inner layer of the membrane, and polyvinyl alcohol/chitosan (PVA/CS) were combined as the outer layer of the membrane to attain the hybrid material PVA/Ag-MOFs @CS (MOF-**16**). The outer layer of the double-layered dressing a has good biocompatibility, while the inner layer has a high antibacterial activity and avoids direct contact with cells. The MOF-**16** possessed an antimicrobial activity against *S. aureus* and *E. coli*, which represent the Gram-positive hybrid materials of Ag-1,3,5-benzenetricarboxylic with *S*- and *N*-carbon quantum dots (CQDs). The MOF-**17** possessed a great antibacterial activity against the representative Gram-positive (*Bacillus subtilis* (*B. subtilis*)) and the Gram-negative (*E. coli*) bacterial strains. The composite formation fostered a synergistic effect that enhanced their antibacterial activity, compared to their new components. The ZOI of *E. coli* and *B. subtilis* is 11–15 nm, and 9.5–13.5 nm, respectively. In addition to the release of metallic silver, the bactericidal activity was linked mainly to the surface charge of the CQDs. These materials interacted with the surface of the bacterial cells through electrostatic forces and thus disrupted the integrity of the cell membrane, leading to bacterial death [115].

Another MOF with the formula {[Ag_6_(μ_3_-HMNA)_4_(μ_3_-MNA)_2_]^2−^·[(Et_3_ NH)^+^]_2_·(DMSO)_2_·(H_2_O)} (AGMNA) (2-thio-nicotinic acid (H_2_MNA)), was incorporated in the polymer hydrogel using, *p*-hydroxyethyl-methacrylate (pHEMA), attaining the MOF-**18** (pHEMA@AGMNA-1). The MOF-**18** possessed the antibacterial capacity against the Gram-negative and Gram-positive bacterial strains. A silver-MOF (AGMNA) enhanced the antibacterial performance more than that of pH EMA. The antimicrobial properties of the silver-containing compounds are due to (i) their interactions with the bacterial cell wall, (ii) their interactions with DNA, enzymes, and membrane protein, and (iii) the generation of the reactive oxygen species [116].

**Figure 4:**
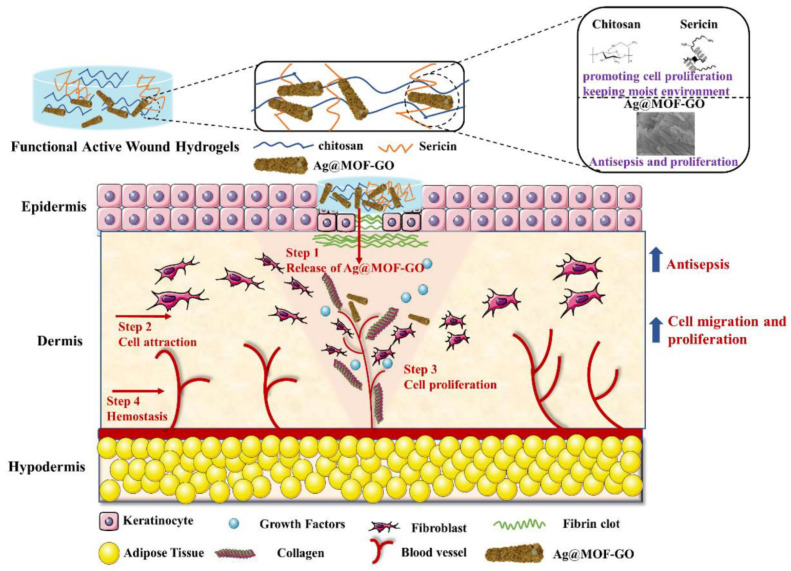
The design of chitosan/silk sericin (CS/SS) hydrogels incorporated with silver nanoparticles @ organic frameworks/graphene oxide (Ag @MOF-GO). Reproduced with permission from [120].

The MOF-**21**, CS/SS/Ag @MOFs-GO, is a silver (Ag)-based metal-organic framework (Ag-MOF) that is embellished with graphene-oxide (GO), whose biocidal activity is higher than those of the Ag-MOFs and the GO nanomaterials. The Ag @MOF–GO impregnated into sericin/chitosan (SS/CS) hydrogels are successfully synthesized through a green strategy. These materials have special properties in improving the cell adhesion, antibacterial activity, biocompatibility, water retention, and antioxidants. The MOFs possess dual antibacterial effects, which contain GO and Ag-MOFs. The Ag@ MOF-GO continuously released the Ag^+^ to the surrounding environment, which caused an interaction between the Ag^+^ and the thiol group protein to destroy the integrity of the bacterial membrane. The interaction between the *O*-containing functional groups of CS/SS/Ag @MOFs-GO and the bacterial lipopolysaccharide promoted the interaction between the Ag^+^ and bacteria to destroy the bacterial cells. In addition, the GO’s antibacterial properties originate from the physical and chemical interaction between the GO and bacterial cell membranes. The sharp edges of the GO damage the bacterial cell membranes [117]; the oxidative stress of the GO damages *E. coli* cells [118]; the GO can generate superoxide anions to damage the bacteria’s cellular membrane [119,120] (Figure 4).

Table 1 reports all of the relevant data obtained from antibacterial tests, in the case of the hybrid Ag-MOFs **13**–**23**, with their antibacterial effect and the mechanism related to each MOF. Table 1 shows that the hybrid silver material had an excellent antibacterial activity against the Gram-negative and Gram-positive bacteria strains, with a preferentially greater activity in the case of the Gram-negative strain. This is due to their cell-wall structural differences with the thick peptidoglycan layer protecting the plasma of the membrane. as the MOFs-**14**, **15**, and MOF-**19** were modified by the TFC membrane to form the nanocomposite film, to improve the antibacterial properties, the water penetration, and the salt selectivity of the film. Those materials possessing superior antibacterial properties were probably related to the release of the Ag^+^ into the solution and the destruction of the framework, the Ag^+^ interacting with lipotropic acid, and the hydroxyl groups of the peptidoglycan membranes.

The antibacterial effect of hybrid the Ag-MOFs not only comes from the release of Ag but also has a certain relationship with the hybrid material. The Ag-MOFs have multiple effects by transferring some material with other effects (such as targeting, increasing stability, biocompatibility, and antibacterial effects) onto MOFs. In the MOF-**17**, the GO has a certain antibacterial ability, and incorporating it with the Ag-MOFs can significantly achieve the antibacterial effect. In addition, we can not only modify some materials on Ag-MOFs but we can load some antibacterial drugs on the MOF to increase its antibacterial spectrum for a better antibacterial effect. The MOFs-**13** and **24** both have a good antibacterial effect on the traditional Gram-positive and Gram-negative bacteria and have a certain therapeutic effect on *MRSA* and other drug-resistant bacteria. The mechanism may be related to the release of the Ag ions, damage to the cell membrane integrity, interfering with the intracellular metabolism of the bacteria, and inhibiting the biofilm formation. Their antibacterial ability comes from the release of silver ions and the action of the antibacterial drugs themselves, which provides a greater practical value for the clinical application.

Simultaneously, the Ag-based material would release the Ag^+^ and diffuse into the interior cytoplasm of the cells to interact with lipotropic acid, the hydroxyl groups of the peptidoglycan membranes, and the phosphate groups of phospholipid membranes [121,122]. Meanwhile, the probable interaction of the Ag(I) ions with DNA and the thiol groups of proteins may damage enzymes and disrupt the integrity and permeability of the bacteria and the catalytic production of ROS [123].

**Table 1:** Antibacterial silver-based MOFs and their composition on the bacterial strain and antibacterial mechanism.

No.	Composition	Organic Ligands	Bacterial Strain	Test Value	Antibacterial Mechanism	Ref.
Pure Ag-MOFs						
**1**	Ag_6_(m-O_3_PC_6_H_4_CO_2_)_2_	*m*-Phosphonobenzoic acid	*S. aureus* *P. aeruginosa*	MBC = 50–70 µM MBC = 20–30 µM	The consequent release of silver ions.	[98]
**2**	[Ag_2_(Cedcp)]_n_	N-(carboxyethyl)-(3,5-dicarboxyl)-Pyridinium bromide	*E. coli* *S. aureus*	MIC >37.84 µM MIC = 37.84 µM	1: The synergistic effect of the aromatic ring and pyridine. 2:The release of Ag^+^.	[99]
**3**	[Ag_2_(μ_3_-PTA)_2_(μ_2_-chdc)]_n_·5nH_2_O	1,3,5-triaza-7-Phosphaadamantane	*S. aureus* *E. coli* *P. aeruginosa*	MIC = 10 µg mL^−1^ MIC = 7 µg mL^−1^ MIC = 6 µg mL^−1^	The release of Ag^+^.	[100]
**4**	[Ag_2_(μ_4_-PTA)(μ_4_-mal)]_n_	1,3,5-triaza-7-Phosphaadamantane	*E. coli* *P. aeruginosa* *S. aureus*	MIC = 7 µg mL^−1^ MIC = 6 µg mL^−1^ MIC = 8 µg mL^−1^	The weak binding tendency of O and N donor atoms toward the center helps the slow release of Ag(I).	[101]
**5**	[Ag_4_(µ-PTA)_2_(µ_3_-PTA)_2_(µ_4_-pma)(H_2_O)_2_]_n_·6nH_2_O	1,3,5-triaza-7-Phosphaadamantane	*E. coli* *P. aeruginosa* *S. aureus*	MIC = 5 µg mL^−1^ MIC = 5 µg mL^−1^ MIC = 8 µg mL^−1^	Bond strengthens between Ag(I) and the ligand donor atoms and the Ag^+^ release.	[102]
**6**	[Ag(u_3_-PTA=S)] _n_(NO_3_) _n_·nH_2_O	1,3,5-triaza-7-Phosphaadamantane-7-sulfide	*E. coli* *P. aeruginosa*	MIC = 4 µg mL^−1^ MIC = 5 µg mL^−1^	Presence of silver nodes.	[103]
**7**	[Ag_4_(u_4_-PTA=S)(u_5_-PTA=S)(u_2_-SO_4_)_2_(H_2_O)_2_]_n_·2nH_2_O	1,3,5-triaza-7-Phosphaadamantane-7-sulfide	*E. coli* *S. aureus*	MIC = 20 µg mL^−1^ MIC = 40 µg mL^−1^	Presence of silver nodes.	[103]
**8**	Ag_5_(PYDC)_2_(OH)	Pyridine-3, 5-dicarboxylic acid	*E. coli* *S. aureus*	MIC = 10–15 ppm MIC = 15–20 ppm	1: The Ag^+^ interacts with bacteria. 2: The damage to the cell membrane.	[104]
**9**	[Ag_2_(O-IPA)(H_2_O)·(H _3_O)]	5-Hydroxyisophthalic acid	*E. coli*	MIC = 5 µg mL^−1^ ZOI = 11.12 mm	Fastest Ag^+^ release rate and highest equilibrium concentration.	[104]
**10**	[AgL]_n_·nH_2_O	4-Cyanobenzoate	*S. mutans* *F. nucleatum* *P. gingivalis*	GIB = 5.29 ppm GIB = 5.29 ppm GIB = 5.29 ppm	Sustained-release of Ag^+^.	[105]
**11**	Ag (NDI-1)_0.5_(H_2_O)	Naphthalenediimide	*E. coli* *S. aureus*	IR = 100% IR = 99.52%	The synergistic reaction of the organic radical and the silver cation.	[106]
**12**	Ag_7_(NDI-2)_1.5_(CH_3_S)_4_(DMSO)3(DMSO)	Naphthalenediimide	*E. coli* *S. aureus*	IR = 99.96% IR = 100%	The synergistic reaction of the organic radical and the silver cation.	[106]
Hybrid Ag-MOFs						
**13**	PLT@ Ag-MOF-Vanc	2-Methylimidazole	*MRSA*	MIC = 0.5 µg mL^−1^	1: Interfering with the intracellular metabolism of bacteria. 2: Catalytic production of the ROS. 3: Damage to the cell membrane integrity.	[111]
**14**	Ag-MOF @TFN	2-Aminoterephthalic acid	*E. coli*	MR = 90–96%	The release of Ag^+^.	[112]
**15**	Ag-MOF/TFC	2-Aminoterephthalic acid	*P. aeruginosa*	MR ~100%	The release of Ag^+^.	[113]
**16**	PVA/Ag-MOF @CS		*S. aureus* *E. coli*	ZOI = 12.1 mm ZOI = 9.7 mm	The release of Ag^+^.	[114]
**17**	CQDs @Ag-MOF	1,3,5-Benzenetricarboxylic acid	*E. coli*	MIC= 4 µg mL^−1^	1: Nanocomposite interactions with the cell membrane. 2: Degradation of the composite material. 3: The release of Ag^+^.	[115]
**18**	{[Ag_6_(μ_3_-HMNA)_4_(μ_3_-MNA)_2_]^2−^·[(Et_3_NH)^+^]_2_·(DMSO)_2_·(H_2_O)}	2-Thio-nicotinic acid	*P. aeruginosa* *S. epidermidis* *S. aureus*	ZOI = 14.0 ± 1.1 mm ZOI = 11.3 ± 1.3 mm ZOI = 11.8 ± 1.8 mm		[116]
**19**	GO−Ag-MOF TFN	1,3,5-Benzenetricarboxylic	*E. coli*	ER: 95%	The synergistic effect of the release of Ag ^+^ and the GO.	[117]
**20**	GO-Ag-MOF	1,3,5-Benzenetricarboxylic	*E. coli* *B. subtilis*	MIC = 50 ppm MIC = 50 ppm	The ROS of the GO damages the bacteria. The release of Ag^+^.	[117]
**21**	CS/SS/Ag- MOF–GO	1,3,5-Benzenetricarboxylic	*S. aureus* *E. coli*		Synergistic effect of the composite GO and the continuously released Ag.	[121]
**22**	P-CS @Ag-MOF	Pyridine-3, 5-dicarboxylic acid	*S. aureus* *E. Coli*	ZOI = 7.82 mm ZOI = 4.32 mm	1: The disruption of cells. 2: Ag(I)interaction with thiol proteins. 3: The combination between the bacterial cell cations and the organic linkers. 4: The release of the ROS.	[122]
**23**	P-CS @Ag- MOF	Pyridine-3, 5-dicarboxylic acid	*S. aureus* *E. Coli*	ZOI = 4.45 mm ZOI = 3.76 mm	1: The disruption of cells. 2: Ag(I) interaction with thiol proteins. 3: The combination between the bacterial cell cations and the organic linkers. 4: The release of the ROS.	[122]
Silver-containing polymer @MOFs						
**24**	SD@Ag@CD-MOF	Cyclodextrin	*E. coli* *S. aureus*	MIC = 4 µg mL^−1^ MIC = 4 µg mL^−1^	The synergistic activity of the releasing Ag^+^ ions and SD.	[124]
**25**	Ag-Phy@ZIF-8@HA	2-Methylimidazole	*S. aureus* *E. Coli*	MIC = 0.13 µg mL^−1^ MIC = 0.25 µg mL^−1^	The synergistic activity of ZIF-8, Ag ^+^, and Phy.	[125]
**26**	Ag-GOD@ ZIF-HA	2-Methylimidazole	*E. Coli* *S. aureus*	MIC = 39.7 µg mL^−1^ MIC = 79.3 µg mL^−1^	Synergetic effect of the release of Ag^+^ and GOD.	[126]
**27**	Ag-NPs@Ni-MOFs	Di-topic carboxylate	*E. Coli* *P. aeruginosa*	MIC = 0.025 ìg/mL MIC = 0.025 ìg/mL	Synergetic effect of the release of Ag^+^ and Ni-MOF.	[81]
**28**	Poly Cu-MOF@ Ag	Poly(terephthalic acid)	*E. coli* *S. aureus*	MIC = 2–5 µg mL^−1^ MIC = 10 µg mL^−1^	1: Release of Ag^+^ and Cu^2+^. 2: Generation of the ROS.	[127]
**29**	Ag-MIL-101(Cr)	Ditopic terephthalic acid	*E. coli* *P. aeruginosaand* *S. aureus*	MIC = 1ug mL^−1^ MIC = 1ug mL^−1^ MIC = 1ug mL^−1^	The release of smaller-sized Ag^+^ ions.	[128]
**30**	Ag@MOF-5	1,4-Benzenedicar-boxylic acid	*E. Coli* *S. aureus*	ZOI = 16.05 mm ZOI = 14.62 mm	1: Silver ions hinder the bacterial DNA replication. 2: Nano-silver destroys the cell membrane. 3: Produces the ROS of	[129]
**31**	Ag@Mg-MOF-PVDF	Sebacic acid	*S. aureus*	ZOI = 10 mm	the release of Ag^+^.	[130]
**32**	Ag/Zn-MOF	2-Aminoterephthalic acid	*E. coli* *S. aureus*	ZOI= 11 mm ZOI= 12 mm	1: Slow release of the silver ions. 2: Ag^+^ interacts with the S, O, and N atoms.	[131]
**33**	GS5-CL-Ag@CD-MOF	Cyclodextrin	*E. Coli* *S. aureus*	MIC = 16 µg mL^−1^ MIC = 64 µg mL^−1^	The Ag NPs released.	[127]
**34**	MN-MOF-GO-Ag	Gallic acid	*S. aureus* *E. coli* *P. aeruginosa*		The synergistic reaction of the GO and Ag.	[128]

### 2.3. Silver-Containing Polymer @ MOF

A preferential antibacterial activity of the insoluble silver sulfadiazine has been reached due to the co-delivery of the superfine Ag NPs with solubilized sulfadiazine (SD) using the carrier cyclodextrin metal-organic frameworks (CD-MOFs). The MOF-**24** (SD/Ag@ CD-MOF) critically strengthens the antibacterial effect, which can increase the release of Ag^+^ and SD together to produce a synergistic antibacterial action. The hydrophilic CD-MOF can easily dissolve within exudates in the wound region to release the drug and prevent the aggregation of the nano-silver particles, which can enhance the antibacterial effect. The MIC of the MOF-**24** against *E. coli* and *S. aureus* is 4 and 6 µg·mL^−1^ and the MBC are 8 and 64 µg·mL^−1^, respectively [126].

**Figure 5:**
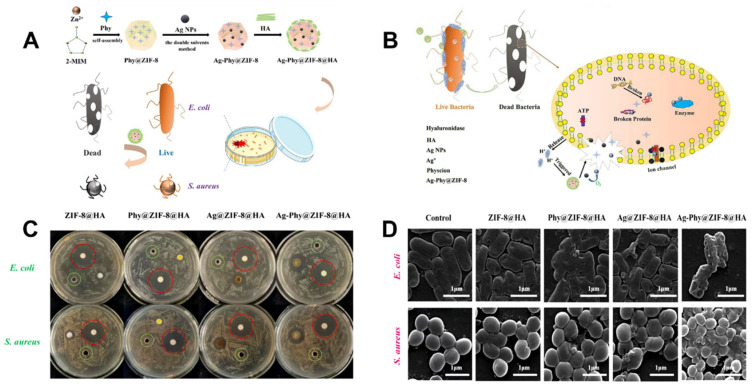
(**A**): Schematic of the Ag-Phy@ZIF-8@HA nanocomposite’s preparation and boosted antibacterial application. (**B**): Possible antibacterial mechanism of the Ag-Phy@ZIF-8@HA. (**C**): Antibacterial circles of *E. coli* and *S. aureus* after they are cocultured with ZIF-8@HA, Ag@ZIF-8@HA, Phy@ZIF-8@HA, and Ag-Phy@ZIF-8@HA for 24 h. Positive control, streptomycin, red circles; Negative control, pH 7.4 PBS, green circles. (**D**): SEM images of *E. coli* and *S. aureus* bacteria strains treated with different nanocomposites, scale bar: 1 μm. Reproduced with permission from [132].

The MOF-**25** Ag-Phy @ZIF-8@HA is a pH-responsive antimicrobial composite nanomaterial prepared by encapsulating the Ag NPs in the ZIF-8, accompanied by the embedding of physcion (Phy). It has an excellent antimicrobial ability against *E. coli* and *S. aureus* (Figure 5C,D). The ZIF-8 possesses the special feature that the Zn^2+^ ion is a beneficial element for humans and the ligands dimethylimidazole is also a good bacterial inhibitor. The ZIF-8 can be disintegrated slowly in a different environment, which is good for releasing drugs and other materials. The antibacterial mechanism was due to the efficient synergistic effect of the ZIF-8, silver, Phy, and the bacterial growth secretes hyaluronidase, which breaks down HA, causing the surrounding environment to become acidified (Figure 5B). The pH response is triggered to cause the crack in the nanomaterial to release the Ag NPs and Phy, which further releases the Ag^+^ ions and destroys the bacterial membrane’s ionic channel and permeability [132].

**Figure 6:**
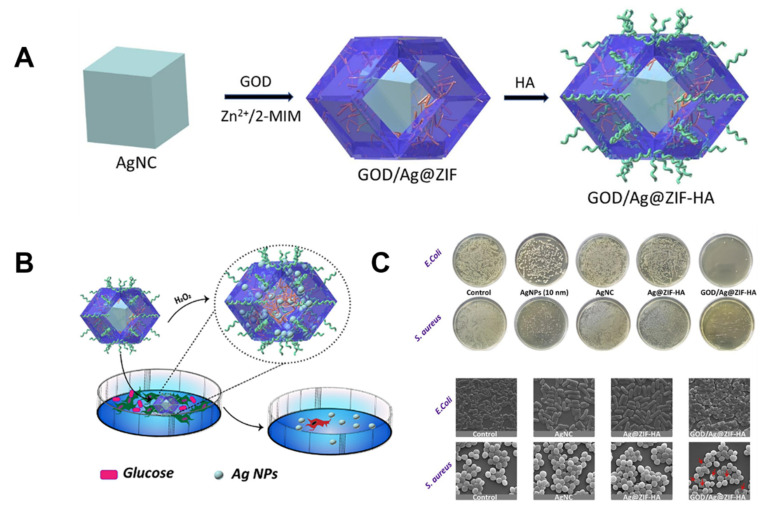
(**A**): Schematic illustration of the preparation of the GOD/Ag @ZIF-HA nanocomposites. (**B**): Schematic illustration of the antibacterial application of GOD/Ag @ZIF-HA. (**C**): Photographs of *E. coli* and *S. aureus* bacteria treated with different nanocomposites at the same Ag concentration (2-fold MIC value and SEM image *E. coli* and *S. aureus* bacteria strains treated with different nanocomposites. Reproduced with permission from [133].

The MOF-**26**, GOD/Ag @ZIF-HA, the controlled encapsulation of a large-size single Ag nano-cube (50 nm) in the zeolitic imidazolate frameworks (ZIFs), accompanied by the embedding of glucose oxidase (GOD), and hyaluronic acid (HA), were coated into the MOF. The obtained MOF-**26** could act as an Ag nano-factory to generate ultrasmall Ag NPs, which is good for releasing the Ag ions. Specifically, the GOD-induced generated H_2_O_2_ in glycolysis continuously decomposed silver into Ag^+^ and ultrasmall Ag NPs, where the biocompatible ZIF-8 acted as the porous support for Ag^+^ and the Ag NP formation. The HA possesses a good biocompatibility and target ability that is good for the MOFs to achieve the antibacterial effect. The MOF-**26** could completely suppress the growth of the two model bacteria strains at low concentrations (*E. coli* for 5 μg mL^−1^, *S. aureus* for 10 μg mL^−1^). The mechanism of MOF-**26** may be related to the particle size of the Ag, the small particle size allowed into the cells and released abundant Ag^+^, which caused the cell death in terms of oxidative stress, the mitochondrial membrane, and the cell cycle progression [133] (Figure 6).

The Ag @Ni-MOF-**27** was prepared by loading silver onto the Ni-MOF. Despite the good antibacterial affinity of the Ni-MOF nanosheets, the MOF-**27** exhibited an obvious antibacterial activity against the four microbes (*B. subtilis*, *E. coli*, *P. aeruginosa*, and *C. albicans*) after 48 h of incubation. It is due to the synergistic effect of the Ni-MOF and Ag cations. The chelation could reduce the polarity of the nickel ions and increase the lipophilicity of the nickel atom, which was beneficial to enhancing the membrane penetration of the MOFs. Meanwhile, the MOFs can act as a reservoir for the metal cations. The released cations can change the microbes’ ionic nature and destroy the ion channels. The released Ag(I) ions can interact with the thiol groups in proteins [134] and can inactivate the respiratory enzymes and disrupt the bacterial membrane integrity and the permeability [124]. Moreover, the metallic Ag can induce oxidative stress on the microbes and damage the membrane [81].

**Figure 7:**
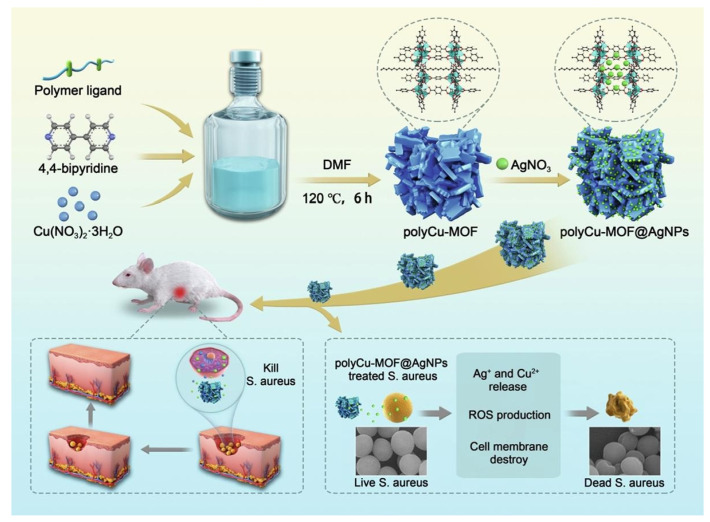
Schematic of the synthesis procedure and the antibacterial and wound healing activities of the poly Cu-MOF@ Ag hybrid. Reproduced with permission from [125].

The copper-based polymer-MOF-**28**, poly Cu-MOF @Ag, is an efficient scaffold loading silver nanoparticle, which was used as a preferential antibacterial agent. The MOF-**28** hybrid contains lower copper nodes and a higher Ag, showing the amount of Ag^+^ and Cu^2+^ ions to enhance the biocompatibility. The MOF-**28** can efficiently kill *E. coli* (MIC value 10 µg mL^−1^) and *S. aureus* (MIC value 10 µg mL^−1^) via damaging the cell integrity by the produced ROS and the disruption of the membrane of the bacteria. The release of the Ag^+^ ions in the presence of the bacterial cells could be increased because the Ag NPs can interact with the sulfur-containing proteins of the cell walls (Figure 7) [125].

The MOF-**29**, Ag-MIL-101 (Cr), was prepared by containing the silver nanoparticles on the MIL-101(Cr). The MOF-**29** have an obvious antibacterial phenomenon against both the Gram-negative (*E. coli* and *P. aeruginosaand*) and Gram-positive (*S. aureus*) bacteria strains, and the MIC of the MOF-**29** to *E. coli*, *P. aeruginosaand*, and *S. aureus* is 1 mg L^−1^. The MIL-101 are a porous organic-inorganic hybrid with a high surface area and a well-defined pore structure. The MOFs can be used to stabilize the Ag metal in an adjustable size. Hence, the antibacterial mechanism may be related to the particle size, the uniform distribution, and the absence of aggregation of the Ag metal. Because of the presence of the MIL-101, the Ag ions are more stable in their structure and can be better released in the application [126].

MOF-5 was prepared using the hydrothermal method, and the Ag@MOF-5 (MOF-**30**) nanoplates were obtained to improve the Ag nanoparticles’ antibacterial and dispersion activities. The antibacterial activity of the MOF-5 has been limited because of the location of the Zn^2+^ ion in the MOF-5 skeleton. However, MOF-**30** showed a better antibacterial activity against *E. coli* (ZOI = 16.05 mm) and *S. aureus* (ZOI = 14.63 mm), which is due to the addition of the silver nanoparticles that make it easier for the metal ions to diffuse into the bacteria, destroy the bacterial membranes and inhibit the cell division [123,134].

**Figure 8:**
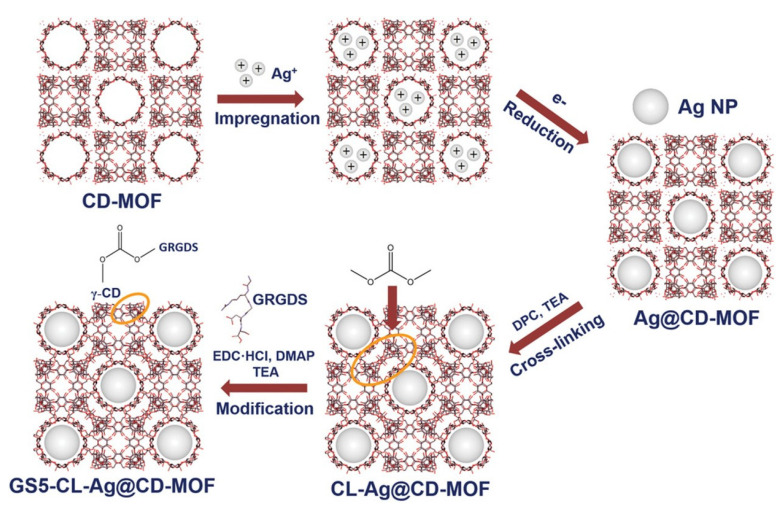
The schematic diagram of the CD-MOF template that guided the synthesis of the Ag NPs by solution impregnation, followed by reduction, cross-linking, and the GRGDS surface modification. Reproduced with permission from [127].

These γ-cyclodextrin metal-organic frameworks (CD-MOFs) possess a good water solubility and biocompatibility, which can act as a template to prepare the Ag@CD-MOFs. These types of materials can achieve a dual function in reducing the particle size and enhancing the stability. Meanwhile, the small silver particles are easily dispersed in the aqueous media and exhibit an effective bacterial inhibition. The GRGDS peptide was modified on the surface of the Ag@ CD-MOFs to generate the MOF-**33**) (GS5-CL-Ag-@CD-MOFs) to enhance the hemostasis to promote the wound recovery and cooperate with the antibacterial effect [127]. The MOF-**33** has an obvious antibacterial effect against *E. coli*. The MIC of *E. coli* is 16 µg mL^−1^. The antibacterial mechanism of the MOF-**33** is the synergistic effect of the CD-MOFs, Ag, and the GRGDS peptide, which means the smaller Ag particle is released from the Ag@ CD-MOF, which might reach immediate contact with the bacterial surface after the dissolution of the CD-MOF template that can increase their bactericidal activity. The peptide is beneficial to wound recovery (Figure 8) [127].

**Figure 9:**
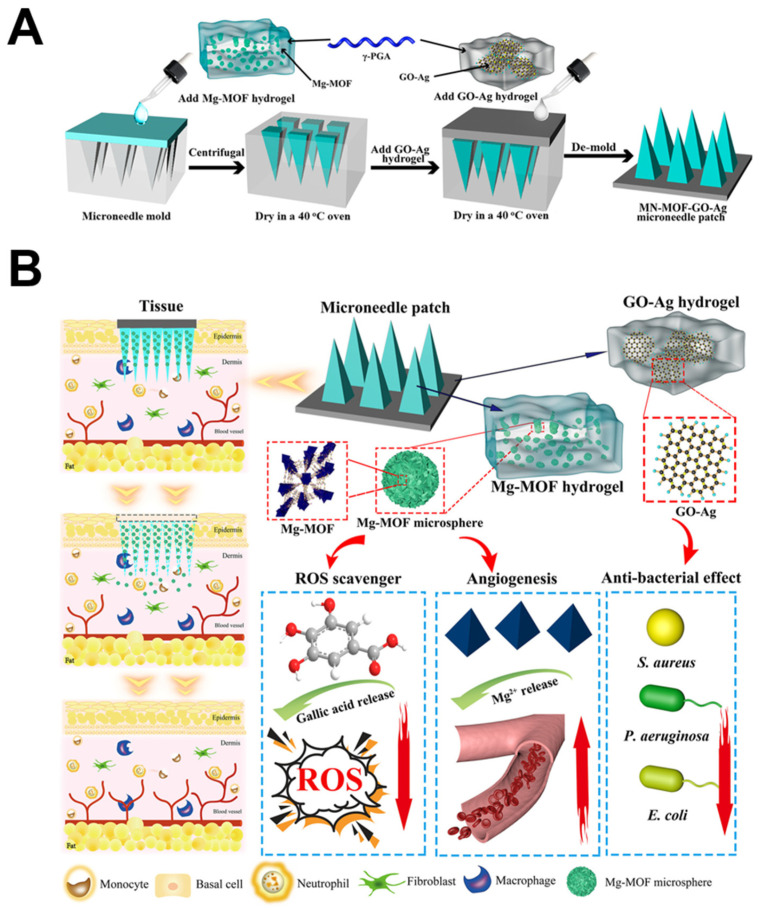
(**A**): Schematic for the MN-MOF-GO-Ag synthesis. (**B**): Illustration of the magnesium organic framework-based MN Patch (Denoted as MN-MOF-GO-Ag) for the accelerated diabetic wound healing. Reproduced with permission from [128].

The Mg-based MN-MOF-GO-Ag (MOF-**34**) was prepared, and the graphene oxide-silver nanocomposites (GO-Ag)-loaded poly (γ-glutamic acid) (γ-PGA) hydrogel was used as the backing layer of the microneedle (MN) patch. Meanwhile, the γ-PGA hydrogel loaded with the Mg-MOF was fabricated into the MN tips to achieve the controlled and long-termed release in the dermis. The Mg-MOFs have a lower cytotoxicity than the Cu. The gallic acid of MOF-**34** can scavenge the overproduced intracellular reactive oxygen species. The GO-Ag can inhibit the foreign body accumulation and accelerate the healing process (Figure 9) [128].

In Table 1, we have tabulated relevant data from the antibacterial activity with the silver-containing polymers @ MOFs **24**–**34**. We have also explored their composition and the antibacterial mechanism of each MOF with other parameters. The silver or silver-containing compounds are loaded into other MOFs to form silver-containing polymers that exploit better the antibacterial properties. The antibacterial mechanism associated with such MOFs is possibly due to the synergistic action of releasing the Ag^+^ ions and compounds. The metallic silver ions are released from the frame system and adhere to the bacterial membrane; the Ag accumulation in the cell membrane affects its permeability [134].

The MOFs-**24**, **25**, **27** and **29** exhibit a great antibacterial activity for the Gram-positive and Gram-negative bacterial strains, those compounds all use the MOFs as a repository, constantly releasing silver ions and the destruction of the framework and then penetrating the bacteria inside and the indirect production of the ROS by the Ag^+^. At the same time, these silver-containing polymers have an attractive antibacterial activity, due to the synergistic effect of the MOFs and the silver cations.

The MOF-**27** contains the Ni atom, which is a chelation reducing the polarity of the nickel ions. Moreover, the free organic moieties of the MOFs can bind with Ca(II) and Mg(II) of the microbial cells and the Ag ions, interacting with the thiol groups of proteins, inducing oxidative stress on the microbes and damaging the membrane [134,135]. The ZIF-8 and MIL-101 in MOFs-**25** and **29** have a higher porosity, which is good for encapsulating silver and can release silver slowly to achieve the long-term antibacterial effect. The MIL-101 can control the particle size of the silver and make the silver ions fully dispersed, which is conducive to the greater antibacterial effect of the silver in the treatment process. The Zn^2+^ ions can exert an antimicrobial activity against the bacterial strains, which is related to their ability to disrupt the cell membrane by the electrostatic interaction (zinc ions) or the generation of the reactive oxygen species (ROS) (zinc oxide), but also to bind to proteins and DNA, inactivating their functions and to modify the expression of several genes.

In the MOFs-**25** and **26**, other compounds also provide excellent conditions for the antibacterial effects, in addition to the zinc and silver metal ions. The HA possesses good hydrophilic and biodegradable properties, enhancing the bacterial-targeted drug release and reducing many side effects from the antibiotic drugs. The GO also plays a role in the antibacterial, synergistic effect of both the physical and chemical aspects. For example, the GO edge causes cell damage, which can directly interact with the cells and lead to the loss of integrity of the cell membrane; the production of the ROS and the transfer of charge; the dissociation of the oxygen-containing groups of the GO, which causes a decrease in the pH of the bacterial microenvironment.

## 3. Molecular Docking

**Figure 10:**
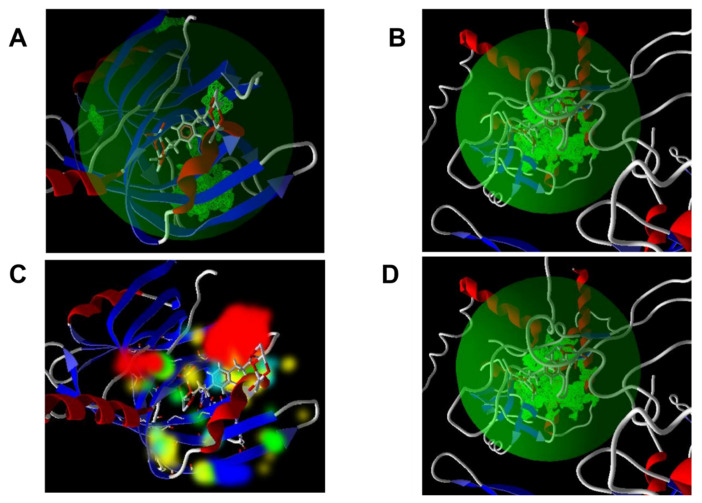
The potential ligand binding cavity of (**A**) *E. coli* and (**B**) *S. aureus* with flexible residues. Energy map of the ligand at the binding cavity of the (**C**) *E. coli* and (**D**) *S. aureus* proteins. Reproduced with permission from [129].

Molecular docking can be used to predict the affinity and binding orientation of the drugs to their biotargets, such as proteins. In the article of Maryam Aghaee et al., the molecular docking experiment was used to prove that the Ag MOF has a certain effect on the antibacterial activity. The silver (I) metal-organic coordination polymer [Ag_2_ L]_n_ (H_2_ L=1,4-phenyl-enedipropionic acid) was fabricated using a sono-chemical technique [129]. [Ag_2_ L]_n_ is appropriately coordinated against the Gram-positive and Gram-negative bacteria, which means that it has an obvious antibacterial effect on both kinds of bacteria. The MIC of [Ag_2_ L]_n_ against *E. coli* and *S. aureus*, is ranged 0.06–0.125, 0.25 (ug/mL), respectively. The result of the molecular docking implies a favorable ligand-protein interaction energy at the binding cavity of the *E. coli* and *S. aureus* proteins. Both the Gram-positive and Gram-negative bacteria have potential ligand binding cavities, as shown in (Figure 10), which indicates that the active site in the MOF can realize the ligand-protein interaction in the ligand binding cavities of the two bacteria.

**Figure 11:**
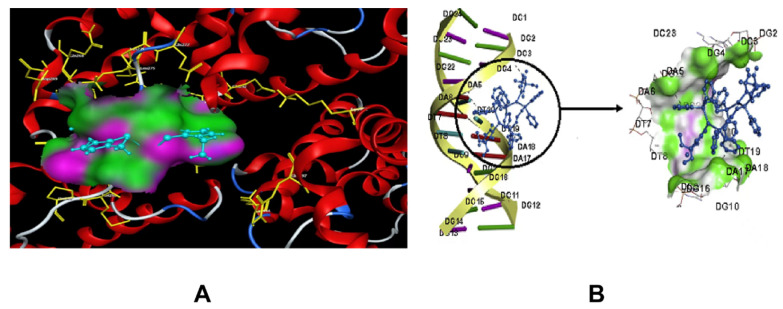
(**A**): The hydrogen bond (violet) and the hydrophobic interactions (green) between the cationic moiety of the asymmetric unit of polymer 1 and HSA. (**B**): Molecular docked structures of [Ag_2_(barb)2(u-dppp)_2_] with DNA (barb = 5,5-diethylbarbiturate), (dppm = 1,1-bis(diphenylphosphino)methane). Reproduced with permission from [104,135].

In the article by Azizolla Beheshti et al., the binding mode and the intermolecular interactions of the bbit and the cationic moiety of the asymmetric unit of the polymer {[Ag(u_2_-bbit)](BF_4_)}_n_ (polymer 1) (bbit=1,1′-(butane-1,4-diyl)bis(3-methylimidazoline-2-thione) ) with HSA (Human serum albumin), was investigated by the molecular docking method [135]. As shown in the (Figure 11A), multiple C atoms in the bbit interact with multiple amino acids in the HSA by the hydrophobic interactions and the hydrogen bonding interactions, making the coordination polymer more stable. It is also indicated in Veysel T. Yilmaz’s opinions, that the interaction between the complexes and HSA is mainly dominated by the hydrophobic and electrostatic interactions. From the docked structures presented in (Figure 11B), the Ag coordination polymers bind reasonably well to DNA through the partial intercalation favored in the grooves of the DNA rich in G/C bases [97].

The molecular docking simulation experiment further shows that the antibacterial effect of the Ag-MOFs are not only to combine with the potential coordination cavity of the bacteria itself, but also to effectively combine with DNA to produce an interaction to achieve the best antibacterial effect. We can further study the specific antibacterial mechanism of the Ag MOF through the relevant ligand groups or active sites displayed by the molecular docking. As far as we know, antibacterial silver mainly refers to the high conductivity of the silver ions, which can attach to the cell membrane and damage the integrity of the membrane, causing the leakage of substances inside the membrane, DNA replication damage, etc.

## 4. Conclusions and Challenges

The most severe threat to the public’s health is a pathogenic bacterial infection. At one time, the use of antibiotics allowed us to fight all kinds of bacterial infections effectively. However, the lack of understanding of antibiotics and people without knowing about the development of “bacterial resistance” led to the emergence of the current “superbugs”. Over the last few decades, numerous efforts have been invested in solving these problems, and various antibacterial materials have been developed. In this perspective, we have focused on the antibacterial effect and mechanism of the Ag-MOFs and the Ag-containing polymer @MOF material as new anti-infective materials. The persistent antibacterial activity with an obvious antibacterial effect, a high stability, a low cost, and a low toxicity are the necessary characteristics of the Ag-MOFs in practical application.

On the antibacterial side, the Ag-MOFs express a superior antibacterial efficiency against the Gram-positive and Gram-negative bacteria strains, with a greater effect on the Gram-negative bacteria. This is due to their cell wall structural differences with the thick peptidoglycan layer protecting the plasma membrane [136,137,138]. It was found that, by comparing of the evidence from the literature, on Ag-MOFs with different assembly structures, that the antibacterial properties of the Ag-MOFs are mainly related to (i) the release of the Ag^+^ ions, (ii) the accumulation of the Ag^+^ on the bacterial membranes destroying the integrity of ion channels and membranes, (iii) the Ag^+^ ions react with the thiol groups of protein, DNA, enzymes, etc., (iv) the ROS production can also lead to bacterial death, (v) the synergistic reaction between the organic linkers and the MOFs (Ag-based or other metals).

The Ag-based MOFs with the special skeleton of the three-dimensional structure enhance the stability of the silver materials, which is more conducive to the release of the Ag cation, to reduce the harm of silver metal to human beings and the environment; silver has good antibacterial properties, and its antibacterial and antimicrobial spectrum can be amplified by combining with the MOFs. Compared with the silver compounds or silver nanomaterials alone, the Ag-MOF greatly improves the antibacterial effect; at the same time, the silver has been adopted as an antimicrobial material and disinfectant that is relatively free of adverse effects. However, in practical application, the defects of silver-based materials cannot be ignored. Firstly, the external condition in the synthetic process is difficult to control, the temperature, brightness, and reaction time all affect the yield and quality of the Ag-MOF; a large number of expensive silver reagents can be wasted during the reaction, resulting in a high cost of the synthesis method; in addition, reductants such as borohydride, dimethylformamide, and thiol can produce many by-products, which not only increase the cost of materials and subsequent treatment but also damage the ecological safety and threaten human health [86].

Although Ag-MOFs have made a breakthrough in the antibacterial field, in recent years, further research is needed to explore their antibacterial mechanism and related applications based on the Ag-MOF materials, to apply them in clinical practice. Based on the recent report which we have discussed in this literature review, we reasonably proposed some improved strategies to meet our expectations: (1) Materials that are hybridized with silver should be subjected to toxicological tests, (2) To study the pathway by which silver ions bind to the thiol proteins and destroy the related channels, (3) The Ag-MOFs can better control the release of the Ag^+^ or the drug, (4) Achieve an antibacterial concentration while reducing the toxicity to normal cells.

## Abbreviations

**MOF**	**Metal-Organic Frameworks**
ROS	Reactive Oxygen Species
ZOI	Zone Of Inhibition
CP	Coordination Polymers
MIC	Minimum Inhibitory Concentration
MBC	Minimum Bactericidal Concentration
GIB	Generation Inhibition Rate
SR	Survive Rate
MR	Mortality Rate
ER	Extirpation Rates
IH	Inhibition Halo
IR	Inhibition Rate
GI	Generation Inhibition
HO-H_2_IPA	5-Hydroxyisophthalic Acid
H_2_PYDC	Pyridine-3, 5-Dicarboxylic Acid
L	4-Cyanobenzoate
PTA	1,3,5-Triaza-7-Phosphaadamantane
PTA=S	1,3,5-Triaza-7-Phosphaadamantane-7-Sulfide
TFC	Thin-Film Composite
GO	Graphene Oxide
TFN	Thin-Film Nanocomposite
SD	Solubilized Sulfadiazine
CD-MOF	Cyclodextrin Metal-Organic Frameworks
GOD	Glucose Oxidase
MIL	Materials Of Institute Lavoisier
CQD	Carbon Quantum Dots
CS/SS	Chitosan/Silk Sericin
PVA	Polyvinyl Alcohol
P-CS	P-Coumaric Acid Modified Chitosan
PLT	Platelets
HEMA	Hydroxyethyl-Methacrylate
H_2_ MNA	2-Thio-Nicotinic Acid
Et_3_ N	3-Ethylene-Amine
DMSO	Dimethylsulfoxide
Phy	Physcion
NDI	Naphthalenediimide
chdc	1,4-Cyclohexanedicarboxylic
mal	Malonic
